# Association of Interleukin-17F Polymorphism and Mortality Predictors with the Risk of COVID-19

**DOI:** 10.1155/2022/4761631

**Published:** 2022-10-27

**Authors:** Manal M. El-Desoky, Samar Tharwat, Nora Mostafa, Asem A. Hewidy, Rehab A. Elmorsey, Mona S. Abdelhafez, Amira H. El-Ashry, Mona M. Elhendawi, Aya Ahmed Fathy, Fatma Azzahraa Hisham

**Affiliations:** ^1^Medical Biochemistry and Molecular Biology Department, Faculty of Medicine, Mansoura University, Mansoura, Egypt; ^2^Rheumatology & Immunology Unit, Department of Internal Medicine, Faculty of Medicine, Mansoura University, Mansoura, Egypt; ^3^Chest Medicine Department, Faculty of Medicine, Mansoura University, Mansoura, Egypt; ^4^Medical Microbiology and Immunology Department, Faculty of Medicine, Mansoura University, Mansoura, Egypt; ^5^Clinical Pathology Department, Faculty of Medicine, Mansoura University, Mansoura, Egypt; ^6^Public Health and Community Medicine, Faculty of Medicine, Mansoura University, Mansoura, Egypt

## Abstract

**Background:**

Th-17 cells, a proinflammatory subset of CD4 T lymphocytes, have been suggested as a possible cause of coronavirus disease-19 (COVID-19)-related immunological injuries. The aim of this study was to investigate the relationship between IL-17F (rs763780) polymorphism and the susceptibility to and outcomes of COVID-19 infection and to determine the clinical and laboratory predictors of COVID-19 death.

**Methods:**

This case-control study included 132 COVID-19 patients and 135 healthy age- and sex-matched controls. The participants were tested for IL-17F rs763780 polymorphism via TaqMan-based genotyping and for the expression of IL-17 by enzyme-linked immunosorbent assay. This study also investigated the predictors for COVID-19 mortality.

**Results:**

A non-statistically significant association was observed between IL-17F alleles and genotypes with COVID-19 (*P*=0.309, *P*=0.138, respectively). Moreover, no significant difference in the IL-17F genotypes was observed between non-survivors and survivors (*P*=0.482). In the multivariate analysis, the participants with the following characteristics had 17.7-, 11.2-, 8-, and 17.9-fold higher odds of exhibiting in-hospital mortality, respectively: (1) hypertension, (2) age of >57 years, (3) WBC count of >12.6 × 10^3^/mm^3^, and (4) D-dimer of >0.9 ng/ml. The ROC curve analysis showed that IL-17 at a cutoff point of >46 pg/ml was a perfect discriminator of COVID-19 patients from control subjects (AUC = 1.0).

**Conclusion:**

The findings indicate that the IL-17F H161R variant does not influence the risk of COVID-19. However, the IL-17 level is a perfect discriminator of COVID-19 infection. Hypertension, age of >57 years, white blood cell count of >12.6 × 10^3^/mm^3^, and D-dimer of >0.9 ng/ml are the independent predictors for death among COVID-19 patients.

## 1. Background

COVID-19 is a highly contagious disease caused by the coronavirus 2 causing severe acute respiratory syndrome (SARS-CoV-2). It was first observed in Wuhan (China) in December, 2019, and subsequently spread worldwide with millions of infected cases exhibiting illness that varies from no symptoms or mild infection to severe respiratory tract infections and even deaths. Since then, it has evolved into a pandemic and has given rise to challenging health concerns worldwide [1].

COVID-19 is caused by an inflammatory response that causes an overproduction of proinflammatory cytokines, resulting in a cytokine storm, which is accountable for the rapid progression of the disease and acute lung injury [2]. Th-17 cells, a proinflammatory subset of CD4 T lymphocytes, have been suggested to be implicated in the immune-related damage associated with COVID-19. Most of the inflammatory actions of Th-17 cells are mediated by interleukin-17F (IL-17), which activates a variety of signaling pathways, leading to a wide variety of other cytokine production (e.g., IL-6, IL-1*β*, TNF*α*, G-CSF, GM-CSF, and TGF-*β*) and chemokines (e.g., IL-8 and MCP1) by a range of different alveolar cell types (endothelial cells and macrophages) [3]. Six IL-17 family members have been identified (from IL-17A to IL-17F) [4].

Single nucleotide polymorphisms (SNPs) are a type of genetic variation that has been linked to a variety of host responses, disease susceptibility, and severity [2]. IL-17F has many SNPs, among which is rs763780 (7488 T/C), which encodes IL-17F [5]. This SNP is placed in the IL-17F gene's coding area (position +7488). It is composed of a T⟶C substitution at amino acid 161 that leads to a histidine-to-arginine change (H161R). Because it may attach to its receptor without activating a signal, the H161R variation has been shown to operate as a natural antagonist of the wild-type IL-17F [6]. Moreover, it has been shown to correlate with disease severity and poor survival in several inflammatory diseases [7]. The TT and TC genotypes of the rs763780 locus in the *IL17F* gene were shown to be linked with the prevalence of COVID19 infection (per million) [8]. To the best of our knowledge, this association has not been studied among Egyptian population. In this regard, we hypothesize that genetic variation IL-17F may affect the susceptibility to COVID-19 and increase the mortality rates.

Therefore, the aim of this study is to investigate the relation between IL-17F rs763780 polymorphism and the susceptibility to COVID-19 among Egyptian population and to find clinical and laboratory predictors of COVID-19 mortality.

## 2. Methods

### 2.1. Design

This is a case-control study conducted from January, 2021, to January, 2022. All participants provided a written informed consent (or their first-line relative if the patient was unable to provide it). The study protocol was reviewed and approved by the Institutional Review Board of Mansoura University's Faculty of Medicine in Egypt (code number: R.21.03.1278).

### 2.2. Sample Size

Based on the study of Batur and Hekim [9], the authors found a minor allele frequency (C allele) of 17% in cases versus 12% in controls. At a significance level of 5%, 129 COVID-19 cases and 129 healthy controls are required to conduct a pilot study on 10% of the required sample size for a large-scale study with 95% power to detect these minor allele frequencies. An Online Sample Size Estimator [http://osse.bii.a-star.edu.sg/calculation1.php] was used to calculate the sample size. Based on the work of Peduzzi et al. [10], a total sample size of 267 participants is required to conduct a multivariable logistic regression analysis involving eight predictors of in-hospital mortality with an expected frequency of 30% among severe cases [11].

### 2.3. Patients

The case group included 132 COVID-19 patients admitted to Mansoura University Isolation Hospital in either intensive care units or general wards. The inclusion criteria included the following: (a) age more than 18 years old, (b) confirmed laboratory (PCR with a deep nasal swab) and radiologically to have COVID 19 infection, and (c) high resolution CT chest suggestive of CORAD “5.” The control group included 135 age- and sex-matched healthy individuals, not suspected clinically, with normal laboratory findings. Patients aged less than 18 years, pregnant females, and suspected patients who were not confirmed by PCR have been excluded.

### 2.4. Clinical and Laboratory Data

The patients' demographic data were recruited including age and sex. Other relevant clinical and laboratory data were assessed and reported. The severity of cough was evaluated using the cough scoring system (CSS) [12]. The CSS is a two-part questionnaire that addresses symptoms during the day and at night. Cough symptoms were evaluated from 0 to 5, with 0 indicating no cough and 5 indicating the most severe cough, based on the frequency, intensity, and impact of cough on daily activities and sleep. Additionally, the severity of dyspnea was determined using the modified MRC scale [13], which ranges from 0 to 4. Then, the clinical severity was defined according to the World Health Organization (WHO), 2021 [14].

The severity of the lung infection was determined in accordance with the study of Bernheim et al. [15]; each of the five lobes of the lung was examined in chest HRCT scans, scored for the degree to which it was affected, and then categorized as follows: score 0, no involvement (0 percent affected); score 1, minimal involvement (1–25 percent); score 2, mild involvement (26–50 percent); score 3, moderate involvement (51–75 percent); and score 4, severe involvement (76–100 percent). After adding up the results for each of the five lobes, a total severity score was generated, with a possible range of 0 to 20. A score of 1–5 was considered to be “minimum,” 6–10 is considered to be “mild,” 11–15 is considered to be “moderate,” and 16–20 was considered to be “severe.”

### 2.5. Blood Sampling

A volume of 3 ml peripheral blood was collected from each participant and divided into two aliquots. The first aliquot was collected in EDTA and stored at −80°C for DNA extraction, while the second aliquot was used to obtain serum and stored at −20°C until assay for IL-17 concentration. The blood samples were collected from patients prior to the start of any antiviral treatment (e.g., favipiravir) or immunomodulators (e.g., corticosteroids).

### 2.6. Laboratory Measurements (Determination of Serum IL-17 Concentration)

An enzyme-linked immunosorbent assay (ELISA) was used to evaluate the IL-17 expression levels of sixty patients and thirty controls from the test population (for each genotype) in accordance with the manufacturer's instructions (INNOVA BIOTECH CO., LTD., Catalog No: In-Hu2141).

### 2.7. DNA Isolation and Genotyping

In accordance with the manufacturer's instructions, genomic DNA was isolated from whole blood using the Quick-DNATM Miniprep Kit (ZymoResearch, USA, Cat. NoD3024). Nanodrop (Thermo Scientific, Wilmington, DE) was used to assess the quality and quantity of the DNA; a result of 1.8–2 was considered a good quality.

The participants were genotyped for rs763780 SNP via TaqMan allelic discrimination assay. It was made up of primers and two probes labeled with a fluorescent dye (VIC and FAM) with the following sequence (VIC/FAM) : GTGGATATGCACCTCTTACTGCACA[C/T] GGTGGATGACAGGGGTGACGCAGGT. All PCR reactions were performed with a total volume of 20 *μ*l of 20 ng genomic DNA, 10a0*μ*l TaqMan Master Mix (Thermo Fisher Scientific, catalog no. 4371353), 1a0*μ*l Taq-Man Genotyping Assay (Thermo Scientific, Catalog number: 4351379), and nuclease-free H_2_O.

The PCR reaction was performed using the StepOne Real-Time PCR system (Applied Biosystems, Foster City, CA, USA) with thermal cycling protocol as follows: 95°C for 10 min and then 40 cycles of 95°C for 15 sand 60°C for 1 min. Randomly selected PCR products were genotyped multiple times for quality control, and the results were 100% consistent.

## 3. Statistical Analysis

The IBM-SPSS software (IBM Corp., released 2019) was used to evaluate the data. Chi-square test was used to compare qualitative data expressed as *n* (%). COVID-19 cases were assigned to two groups according to in_hospital mortality (non-survivors and survivors). For the two groups, quantitative data were expressed as median and interquartile range (IQR) and compared using the Mann–Whitney *U* test. Simple binary logistic regression was used to estimate the crude odds ratios (univariable), odds ratios (multivariable), and their 95% CI. The SNPStats web tool (https://www.snpstats.net/start.htm) was used for SNP study. A *P* value of ≤0.050 was considered statistically significant for any of the used tests.

## 4. Results

### 4.1. Demographic Data and Associated Comorbidities

This study included 267 participants divided into two groups. The patient group involved 132 documented COVID-19 cases, 76 female (57.6%) vs. 56 male (42.4%), with a median age (25th–75th percentiles) of 65 (49.3–71) years. The control group involved 135 healthy individuals, 64 female (47.4%) vs. 71 male (52.6%), with a median age (25th to 75th percentiles) of 57 (47–71) years. The age and sex distributions of the two groups were not significantly different (*P*=0.125 and 0.096, respectively).

At least one preexisting comorbidity was observed in the COVID-19 cases with the most common ones being diabetes mellitus (65.2%, 86/132) and hypertension (72%, 95/132). Moreover, the presence of ≥2 morbidities in the same individual is present in 73.5% (97/132) as shown in Table 1.

### 4.2. Clinical, Laboratory, and Radiological Data

This study enrolled 132 patients with COVID-19 infection: the survivors (*n* = 75) and non-survivors (*n* = 57).  Table 2 shows the clinical, laboratory, and radiological properties of patients.

A statistically significantly higher proportion of age (*P* < 0.001), clinical severity (*P* < 0.001), duration of hospital stay (*P*=0.028), WBCs count (*P*=0.022), CRP (*P*=0.001), D-dimer (*P* < 0.001, and CT severity score (*P* < 0.001) were observed in the nonsurvivors than in the survivors. Moreover, individuals with hypertension and two or more morbidities were significantly higher among non-survivors (*P*=0.006 and 0.042, respectively).

However, no statistically significant difference was observed in the sex (*P*=0.518), fever, cough symptom score (*P*=0.196), hemoglobin concentration (*P*=0.793), lymphocytes (*P*=0.157), and platelets (*P*=0.938) between the non-survivors and survivors. The neutrophil count of the non-survivors was higher than that of the survivors but not statistically significant (*P*=0.055).

### 4.3. Serum Level of IL-17

**Figure 1:**
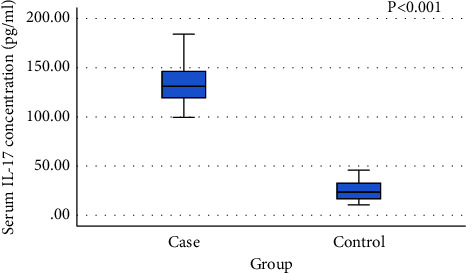
Serum level of IL-17 in the study COVID-19 patients (*n* = 132) and healthy controls (*n* = 135). *P* < 0.001.

**Figure 2:**
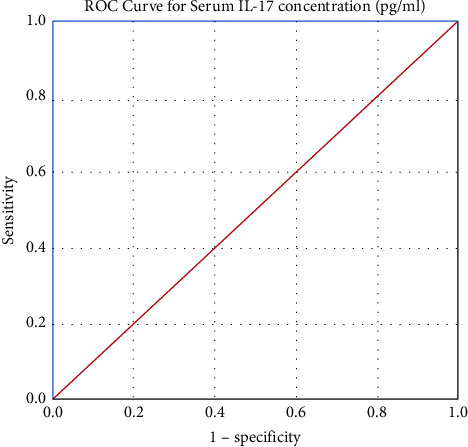
A ROC curve analysis for IL-17 concentration as a discriminator of COVID-19 patients.

The results indicated that the median concentration of IL-17 (pg/ml) (25th–75th percentiles) were 131 [119.2–146.6] in COVID-19 cases vs. 23.5 (16.45–32.7) in control subjects. This difference was considered statistically significant (Z value = −7.703, *P* < 0.001). The ROC curve analysis showed that IL-17 at a cutoff point of >46 pg/ml was a perfect discriminator of COVID-19 from control subjects (AUC = 1.0) (Figures 1 and 2).

### 4.4. Genotype and Allele Frequencies

For IL-17F rs763780, no statistically significant association was observed between IL-17F alleles and COVID-19 (*P*=0.309, *φ* = −0.044). The “C” allele was marginally higher in COVID-19 cases vs. controls (6.4% vs. 4.4%). Moreover, no statistically significant association was observed between IL-17F genotypes and COVID-19 (*P*=0.138, *φ* = 0.091) (Table 3). Also, there was no statistically significant difference observed in the IL-17F genotypes between survivors and non-survivors (*P*=0.482).

### 4.5. Best Inheritance Model

Both the COVID-19 cases and control groups were in the Hardy–Weinberg equilibrium (HWE). The result of the exact test for HWE was statistically insignificant (*P*=1.00 and 0.23, respectively). The overdominant inheritance model was the best inheritance model adjusted for age and gender (with the lowest *P* value (0.067), AIC (279.3), and BIC (293.7)). Participants with the C/T genotype had a 2.5-fold higher chance of exhibiting COVID-19 than those with the C/C or T/T genotypes (adjusted for age and sex).

### 4.6. Predictors of In-Hospital Mortality in COVID-19 Cases

The effects of 11 predictor variables on the likelihood of mortality were investigated using binomial logistic regression. In the analysis, two standardized residuals with values of 2.582 and −2.684 standard deviations were kept.

The logistic regression model, *χ*^2^ (11) = 116.946, *P* < 0.001, was statistically significant. The model accurately identified 90.9% of the cases and explained 78.9% (Nagelkerke *R*2 = 0.789) of the variance in mortality, with a sensitivity, specificity, positive predictive value, and negative predictive value of 93%, 89.3%, 86.9%, and 94.4%, respectively.

Of the 11 predictor variables, presence of hypertension, age of >57 years, WBC count of >12.6 × 10^3^ per mm^3^, and D-dimer of >0.9 ng/ml were all statistically significant independent predictors of the likelihood of death (Table 3). Participants with hypertension, age of >57 years, WBC count of >12.6 × 10^3^/mm^3^, and D-dimer of >0.9 ng/ml had 17.7-, 11.2-, 8-, and 17.9-fold higher odds to exhibit in-hospital mortality as shown in Table 4.

## 5. Discussion

Several studies have been conducted to determine the correlation between genetic variants and the severity or susceptibility of clinical disease to SARS-CoV-2 infection [16]. The molecular mechanisms behind COVID-19 pathology are unknown. However, it is widely believed that COVID-19 is an inflammatory illness and that the onset of inflammation is due to immune regulatory abnormality [17]. In the present study, we investigated the relationship between *IL-17F* rs763780 polymorphism and COVID-19 to verify if this polymorphism could increase the susceptibility to COVID-19 infection.

We observed that no significant association was found between *IL17F* rs763780 polymorphism and COVID-19 and the presence of the minor “C” allele was not significantly associated with a higher risk of COVID-19 infection. This observation was consistent with previous findings [18]. Moreover, no significant association was observed between the risk of mortality and various genotypes. This result suggests that the SNP of rs763780 genetic variants had no impact on the risk and prognosis of COVID-19. In contrast to the existing study, Batur and Hekim [8] discovered a significant positive correlation between the prevalence of COVID-19 and the frequency of the T/T genotype, as well as a negative correlation with the C/T genotype at rs763780. Moreover, they detected a negative relationship between the C/C genotype frequency and mortality rate.

One probable reason is that a single-nucleotide mutation has no effect on the severity of the disease, outcome, or phenotype. To determine the clinical impact of the H161R mutation on COVID-19, more patients must be examined. Other possible contributors, such as other major SNPs, should also be investigated.

In the present study, the IL-17 level was significantly higher in COVID-19 cases compared with controls, as confirmed in previous studies [19,20]. In addition, Huang et al. [21]discovered that COVID-19 patients in the intensive care unit have significantly higher serum levels of IL-17 than controls. Furthermore, blood samples from patients with H_1_N_1_ influenza-induced acute lung damage showed higher levels of IL-17 [22]. Furthermore, IL-17 and other proinflammatory cytokines have been linked to the severity of MERS-CoV, SARS-CoV, and SARS-CoV-2 [23,24]. The inhibition of IL-17 has been widely used for reducing the damage caused by inflammatory autoimmune diseases [25]. These results support the idea that IL-17 inhibitors could be examined as a viable treatment for COVID-19 patients with severe symptoms.

The present study investigated the risk factors for death in COVID-19 hospitalized patients. Hypertension, age of >57 years, WBC count of >12.6 × 10^3^/mm3, and D-dimer of >0.9 ng/ml were the independent predictors for death among patients.

One of the most key factors which influence mortality is age, and in the present study, age of >57 years was an independent factor contributing to mortality. This is consistent with earlier studies, which indicated that the most powerful predictor of death in COVID-19 patients was age [26,27]. Most often, the aged patient revealed a poor response to infection and failed to establish a sufficient protective immunological response [28]. As a result, viral infections (e.g., influenza) tend to be more severe and result in more complications in older people.

Other studies have also stressed the importance of laboratory data in mortality prediction. According to Sisó-Almirall et al. [29], LDH, D-dimer, and CRP were the most critical laboratory indicators related with unfavorable outcomes. Also, Macias-Muñoz et al. [30] showed that low lymphocytic and platelet counts in addition to higher D-dimer, CRP were independent risk factors for death.

However, we observed a significant increase in the WBC count, CRP, and D-dimer in the non-survivors compared with the survivors. Only an increased WBC count and D-dimer level were independent predictors of mortality. Therefore, further research is needed to confirm this.

Comorbidities have been identified as an important risk factor related to increasing severity of the disease [31–33]. Furthermore, various studies have identified a direct relationship between COVID-19 severity and the increase in the number of comorbidities in the same subject [34]. In the same line, our study demonstrated an association between the existence of two or more comorbidities in the same person and mortality. We observed that hypertension increases the risk of COVID‐19 by 3.2‐fold, which is consistent with previous studies [35,36]. Furthermore, in COVID-19 patients, hypertension is considered a predictor of mortality.

With regard to hypertension, the regular use of antihypertensive medications, such as angiotensin II receptor blockers (ARB) and angiotensin-converting enzyme inhibitors (ACEI), elevates ACE2 expression, allowing SARS-CoV-2 to enter pneumocytes, therefore increasing infection severity and mortality [37].

It is important to note that the study has several limitations, including the small sample size and its single-center study design, which may affect gene distribution. Lastly, we looked at a single SNP but this was due to financial support shortage.

## 6. Conclusion

Our findings revealed that the IL-17F H161R variant does not influence the risk of COVID-19 in the studied population. However, IL-17 at a cutoff point of >46 pg/ml was a perfect discriminator of COVID-19 patients from control subjects. Hypertension, age of >57 years, WBC count of >12.6 × 103/mm^3^, and D-dimer of >0.9 ng/ml were the independent predictors for death in COVID-19 patients.

## Figures and Tables

**Table:** 

Sensitivity	100.0
Specificity	100.0
Criterion	>46
AUC	1.000
P	<0.001

**Table 1:** Demographic data and associated comorbidities of the study COVID-19 cases (*n* = 132)

Variable *n* (%), median (25th–75th percentile)	COVID-19 patients total (*n* = 132)	Survivors (*n* = 75)	Non-survivors (*n* = 57)	*P*
Age (years)	65 (49.3–71)	57 (44–68)	69 (60–76)	**<0.001**

*Sex*				
Female	76 (57.6)	45 (60)	31 (54.4)	0.518
Male	56 (42.4)	30 (40)	26 (45.6)	

*Associated comorbidities*				
<2	35 (26.5)	25 (33.3)	10 (17.5)	**0.042**
≥2	97 (73.5)	50 (66.7)	47 (82.5)	

Diabetes mellitus	86 (65.2)	52 (69.3)	34 (59.6)	0.247

Systemic hypertension	95 (72)	47 (62.7)	48 (84.2)	**0.006**

**Table 2:** Clinical, laboratory, and radiological data of the study COVID-19 patients (*n* = 132).

Variable *n* (%), median (25th–75th percentile)	COVID-19 patients total (*n* = 132)	Survivors (*n* = 75)	Non-survivors (*n* = 57)	*P*
*Clinical data*				
Temperature (°C)	38 (37.6–38)	38 (37.7–38)	38 (37.5–38.6)	0.527
RR (breaths/minute)	30 (26–36)	28 (24–30)	35 (30–39)	**<0.001**
Cough symptom score (CSS)	2 (1–2)	2 (1–2)	2 (2–2)	0.196
Modified MRC Dyspnea Scale	3 (3–4)	3 (2–3)	4 (3–4)	**<0.001**

*Clinical severity*				
Noncritical	38 (28.8)	36 (48)	2 (3.5)	**<0.001**
Critical	94 (71.2)	39 (52)	55 (96.5)	
Duration of hospital stay (days)	10 (7–14)	8 (6–13)	11 (8–14)	**0.028**

*Laboratory data*				
Hemoglobin level (g/dl)	11.3 (10.1–12.7)	11.3 (10.1–12.4)	11.3 (10–13)	0.793
Platelet (× 10^3^ per mm^3^)	231 (176.8–282.3)	241 (179–269)	224 (172.5–301.5)	0.938
WBCs (× 10^3^ per mm^3^)	8.9 (6.4–12.9)	7.8 (6.6–11.1)	11.6 (8.5–16.4)	**0.022**
Lymphocyte (× 10^3^ per mm^3^)	1.1 (0.8–1.5)	1.2 (0.85–1.5)	1 (0.7–1.5)	0.157
Neutrophil (× 10^3^ per mm^3^)	6.6 (4.8–11)	5.8 (5.2–9.8)	8.1 (4.4–14.2)	**0.055**
CRP (mg/L)	96 (48–142.8)	88 (40–123)	110 (52.5–170.5)	**0.001**
D-dimer (ng/ml)	0.8 (0.6–1.3)	0.7 (0.45–0.9)	1.2 (0.85–1.5)	**<0.001**

*Radiological data*				
CT severity				
Mild to moderate	38 (28.8)	37 (49.3)	1 (1.8)	**<0.001**
Severe	94 (71.2)	38 (50.7)	56 (98.2)	

*IL-17F genotype*				
C/T	17 (12.9)	11 (14.7)	6 (10.5)	0.482
T/T-C/C	115 (87.1)	64 (85.3)	51 (89.5)	

CRP:C-reactive protein; CSS: cough symptom score (CSS); CT: computerized tomography MRC: modified medical research council; RR: respiratory rate, WBCs: white blood cells.

**Table 3:** IL17 F alleles and genotypes in the stud COVID-19 patients (*n* = 132) and healthy controls (*n* = 135).

IL17F *n* (%)	COVID-19 patients (*n* = 132)	Healthy controls (*n* = 135)	*Chi-square test of association*				*Binary logistic regression*		
			*χ * ^2^	*P*	Phi (*φ*)	*P*	COR	95% CI	*P*
*Alleles*									
“T”	247/264 (93.6)	258/270 (95.6)	1.034	0.309	−0.044	0.309	r (1)	r (1)	0.312
“C”	17/264 (6.4)	12/270(4.4)					1.48	0.69–3.16	

*Genotypes*									
T/T-C/C	115 (87.1)	125 (92.6)	2.198	0.138	0.091	0.138	r (1)	r (1)	0.143
C/T	17 (12.9)	10 (7.4)					1.85	0.81–4.2	

Phi (*φ*): a measure for the strength of association; COR: crude odds ratio; CI: confidence interval. r (1) = reference category.

**Table 4:** Univariate and multivariate logistic regression analyses to identify predictors of mortality in the study COVID-19 patients (*n* = 135).

Variable	*Univariate*			*Multivariate*		
	*P* Value	COR	95% CI	*P* Value	OR	95% CI
*Age (years)*						
≤57	**<0.001**	r (1)	r (1)	**0.002**	r (1)	r (1)
>57		5.1	2.2–11.6		11.2	2.4–53.1

*Blood pressure*						
Normotensive	**0.008**	r (1)	1.4–7.4	**0.013**	17.7	r (1)
Hypertensive		3.2				1.8–172.2

*Associated comorbidities*						
≤2	**0.045**	r (1)	1.02–5.4	0.380	r (1)	r (1)
>2		2.4			0.38	0.04–3.3

*RR (breaths/minute)*						
≤28	**<0.001**	r (1)	4.8–28	0.058	5.9	0.94–36.4
>28		11.5				

*Modified MRC Dyspnea Scale*						
≤3	**<0.001**	r (1)	r (1)	0.8	r (1)	r (1)
>3		6.9	3.1–15		1.2	0.28–4.9

*Clinical severity*						
Noncritical	**<0.001**	r (1)	r (1)	0.057	r (1)	r (1)
Critical		25.4	5.7–111.7		21.1	0.91–487.2

*Duration of hospital stay (days)*						
≤8	**0.008**	r (1)	1.3–5.6	0.381	r (1)	r (1)
>8		2.7			2.2	0.38–12.6

*WBCs (× 10 * ^*3*^ * per mm * ^*3*^ * )*						
≤12.6	**<0.001**	r (1)	r (1)	**0.022**	r (1)	r (1)
>12.6		4.7	2–11		8	1.4–47.7

*CRP (mg/L)*						
≤98	**<0.001**	r (1)	r (1)	0.392	r (1)	r (1)
>98		3.9	1.9–8.2		1.9	0.43–8.7

*D-dimer (ng/ml)*						
≤0.9	**<0.001**	r (1)	r (1)	**<0.001**	r (1)	r (1)
>0.9		12.2	5.3–28.1		17.9	3.1–103

*CT severity*						
Mild to moderate	**<0.001**	r (1)	r (1)	0.116	r (1)	r (1)
Severe		54.5	7.2–414.6		10.7	0.56–206.4

CRP:C-reactive protein; RR: respiratory rate; WBCs: white blood cells.

## Data Availability

The datasets generated during and/or analyzed during the current study are available from the corresponding author on reasonable request.

## Abbreviations

SARS-CoV-2: Severe acute respiratory syndrome coronavirus-2
COVID-19: Coronavirus disease-19
IL-17: Interleukin-17
SNP: Single-nucleotide polymorphism
Real Time-PCR: Real-time polymerase chain reaction
ACE: Angiotensin-converting enzymes
ARB: Angiotensin II receptor blockers
ACEI: Angiotensin-converting enzyme inhibitors
